# Crystal structure of *cis*-aqua­bis­(2,2′-bi­pyridine-κ^2^ *N*,*N*′)chlorido­chromium(III) tetra­chlorido­zincate determined from synchrotron data

**DOI:** 10.1107/S2056989016001870

**Published:** 2016-02-03

**Authors:** Dohyun Moon, Keon Sang Ryoo, Jong-Ha Choi

**Affiliations:** a Pohang Accelerator Laboratory, POSTECH, Pohang 37673, Republic of Korea; b Department of Chemistry, Andong National University, Andong 36729, Republic of Korea

**Keywords:** crystal structure, synchrotron radiation, 2,2′-bi­pyridine, chloride ligand, aqua ligand, *cis*-geometry, chromium(III) complex

## Abstract

The structure of the title salt, [CrCl(C_10_H_8_N_2_)_2_(H_2_O)][ZnCl_4_], has been determined from synchrotron data. The Cr^III^ ion is coordinated by four N atoms from two 2,2′-bi­pyridine (bipy) ligands, one O atom from a water mol­ecule and a chloride anion in a *cis* arrangement, displaying a distorted octa­hedral geometry. The tetra­hedral [ZnCl_4_]^2−^ anion is slightly distorted owing to its involvement in O—H⋯Cl hydrogen bonding with the coordinating water mol­ecule. The Cr—N(bipy) bond lengths are in the range 2.0485 (13)–2.0632 (12) Å, while the Cr—Cl and Cr—(OH_2_) bond lengths are 2.2732 (6) and 1.9876 (12) Å, respectively. In the crystal, mol­ecules are stacked along the *a* axis.

## Abstract 2

The Cr^III^ ion in the title complex is coordinated by two 2,2′-bi­pyridine ligands, one water mol­ecule and a chloride in a *cis* geometry, displaying an overall distorted octa­hedral environment. The slightly distorted tetra­hedral [ZnCl_4_]^2−^ anion is connected to the cation through O—H⋯Cl hydrogen bonds.

## Chemical context   

Chromium(III) complexes with polypyridyl ligands such as 2,2′-bi­pyridine (bipy) or phenanthroline (phen) could be potential candidates as emitting materials in electrochemical cells and sensitizers in dye-sensitized solar cells (Brennan *et al.*, 2008 ▸; Schönle, 2014 ▸). As a prerequisite for possible applications, a detailed study of the structural and spectroscopic properties is needed. Since counter-anionic species also play a very important role in chemistry, pharmacy, biology and environmental process, the mol­ecular recognition of anions or anion binding is an area of current inter­est (Fabbrizzi & Poggi, 2013 ▸; Boiocchi *et al.*, 2014 ▸). Within this context, we report here on the mol­ecular and crystal structure of the title salt, [CrCl(bipy)_2_(H_2_O)][ZnCl_4_], (I).
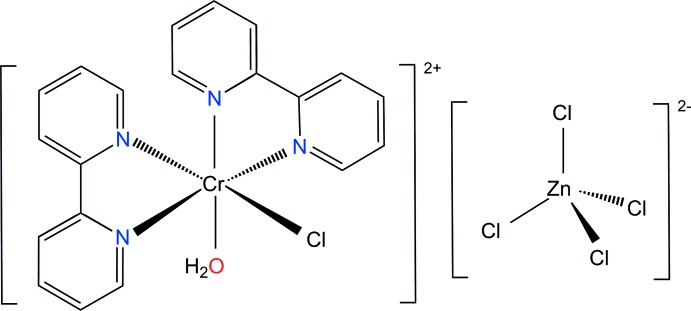


## Structural commentary   

**Figure 1:**
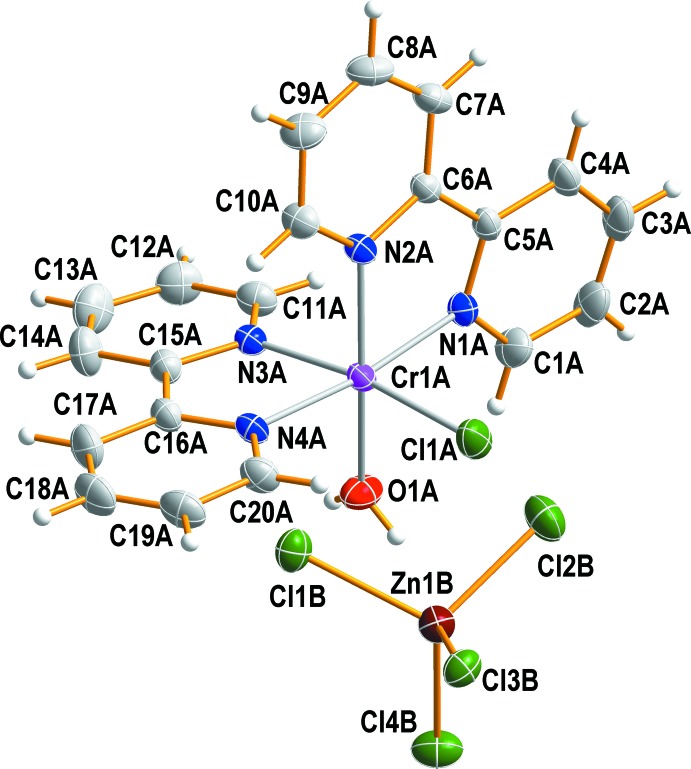
The structure of the mol­ecular components in (I), showing the atom-numbering scheme. Non-H atoms are shown as displacement ellipsoids at the 50% probability level.

In the mol­ecular structure, one chloride anion and one water mol­ecule coordinate to the Cr^III^ ion in a *cis* arrangement, with an O1*A*—Cr1*A*—Cl1*A* angle of 90.13 (4)°. The rest of the coordination sites are occupied by four nitro­gen atoms from two bipy ligands, leading to an overall distorted octa­hedral coordination environment (Fig. 1 ▸). The Cr—N(bipy) bond lengths are in the range of 2.0485 (13) to 2.0632 (12) Å, in good agreement with those determined for *cis*-[Cr(CH_3_COO)_2_(bipy)_2_]PF_6_ (Wang *et al.*, 2013 ▸), *cis*-[CrCl(bipy)_2_(H_2_O)](ClO_4_)_2_·2H_2_O (Wickaramasinghe *et al.*, 1982 ▸) or *cis*-[CrF_2_(bipy)_2_]ClO_4_·H_2_O (Yamaguchi-Terasaki *et al.*, 2007 ▸). The Cr—Cl and Cr—(OH_2_) bond lengths in (I) are 2.2732 (6) and 1.9876 (12) Å, respectively. The latter is comparable to the values of 1.99 (1), 1.9579 (10) and 1.996 (4) Å found in *cis*-[Cr(bipy)_2_(H_2_O)_2_](NO_3_)_3_ (Casellato *et al.*, 1986 ▸), *cis*-[CrF(bipy)_2_(H_2_O)](ClO_4_)_2_·2H_2_O (Birk & Bendix, 2010 ▸) and *trans*-[CrF(3,2,3-tet)(H_2_O)](ClO_4_)_2_·H_2_O (3,2,3-tet = 1,5,8,12-tetra­aza­undeca­ne) (Choi & Lee, 2008 ▸), respectively. The Cr—Cl bond length in (I), however, is slightly shorter than those with 2.289 (9), 2.2941 (15) and 2.3253 (7) Å in *cis*-[CrCl_2_(bipy)_2_](Cl)_0.38_(PF_6_)_0.62_ (Kar *et al.*, 2006 ▸), *cis*-[CrCl_2_(phen)_2_]Cl (Gao, 2011 ▸) and *trans*-[CrCl_2_(Me_2_tn)_2_]Cl (Me_2_tn = 2,2-di­methyl­propane-1,3-di­amine) (Choi *et al.*, 2007 ▸), respectively. The Cl1*A*—Cr1*A*—N3*A* and N1*A*—Cr1*A*—N4*A* angles are 171.51 (5) and 172.67 (5)°, respectively. The bite angles involving the two chelating ligands [N1*A*—Cr1*A*—N2*A* = 79.29 (5) and N3*A*—Cr1*A*—N4*A* = 79.41 (5)°] increase the distortion of the octa­hedral coordination sphere. The Zn^II^ atom in the [ZnCl_4_]^2−^ anion has a distorted tetra­hedral coordination environment due to the influence of hydrogen bonding on the Zn—Cl bond lengths [range: 2.2348 (7) to 2.3127 (6) Å] and the Cl—Zn—Cl angles [range: 103.92 (2) to 112.67 (2)°].

## Supra­molecular features   

**Figure 2:**
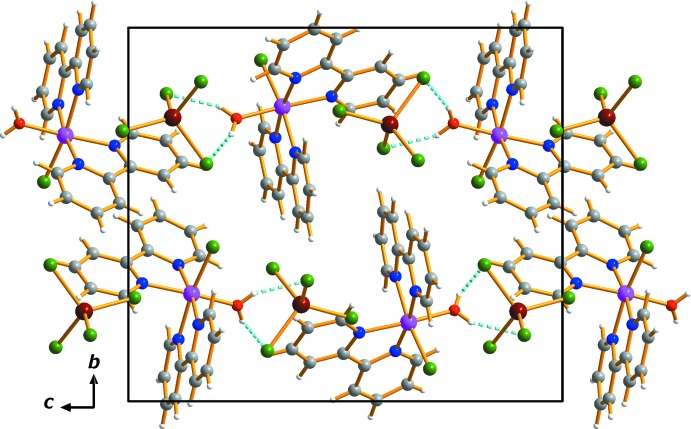
The crystal packing in (I), viewed perpendicular to the *bc* plane. Dashed lines represent O—H⋯Cl hydrogen-bonding inter­actions.

In the crystal, the mol­ecules are stacked along the *a* axis. The supra­molecular set-up involves O—H⋯Cl hydrogen bonds between the coordinating water mol­ecule of the cation as donors and two of the tetra­chlorido­zincate Cl atoms (Cl1*B*, Cl3*B*) as acceptors (Table 1 ▸, Fig. 2 ▸). It is worth noting that the Cl2*B* and Cl4*B* atoms of the [ZnCl_4_]^2−^ anion and the Cl1*A* ligand are not involved in hydrogen bonding.

## Database survey   

A search of the Cambridge Structural Database (Version 5.35, May 2014 with one update; Groom & Allen, 2014 ▸) indicated a total of 18 hits for Cr^III^ complexes containing two bidentate 2,2′-bi­pyridine ligands. The crystal structures of *cis*-[Cr(CH_3_COO)_2_(bipy)_2_]PF_6_ (Wang *et al.*, 2013 ▸), *cis*-[CrCl(bipy)_2_(H_2_O)](ClO_4_)_2_·2H_2_O (Wickaramasinghe *et al.*, 1982 ▸), *cis*-[CrF_2_(bipy)_2_]ClO_4_·H_2_O (Yamaguchi-Terasaki *et al.*, 2007 ▸), *cis*-[CrF(bipy)_2_(H_2_O)](ClO_4_)_2_·2H_2_O (Birk & Bendix, 2010 ▸), *cis*-[Cr(bipy)_2_(H_2_O)_2_](NO_3_)_3_ (Casellato *et al.*, 1986 ▸), *cis*-[Cr(NCS)_2_(bipy)_2_]I_3_ (Walter & Elliott, 2001 ▸), *cis*-[CrCl_2_(bipy)_2_](Cl)_0.38_(PF_6_)_0.62_ (Kar *et al.*, 2006 ▸) and *cis*-[CrCl_2_(bipy)_2_]Cl·H_2_O (Brennan *et al.*, 2008 ▸) have been reported previously.

## Synthesis and crystallization   

All chemicals were reagent grade materials and used without further purification. The starting material, *cis*-[CrF_2_(bipy)_2_]ClO_4_ was prepared according to the literature (Glerup *et al.*, 1970 ▸). The crude perchlorate (0.2 g) was dissolved in 10 mL of 0.01 *M* HCl at 313 K; 0.5 g of solid ZnCl_2_ dissolved in 5 mL 1 *M* HCl were added to this solution. The solution mixture was refluxed for 30 min and filtered. The filtrate was slowly evaporated at room temperature to yield orange crystals of (I) suitable for X-ray structural analysis.

## Refinement   

Crystal data, data collection and structure refinement details are summarized in Table 2 ▸. H atoms were placed in geometrically idealized positions and constrained to ride on their parent atoms, with C—H = 0.94 Å, and with *U*_iso_(H) = 1.2*U*_eq_(C). The H atoms of the water mol­ecule were located from difference Fourier maps and restrained with O—H = 0.84 Å using DFIX and DANG commands (Sheldrick, 2015*b* ▸).

## Supplementary Material

Crystal structure: contains datablock(s) I. DOI: 10.1107/S2056989016001870/wm5266sup1.cif

Structure factors: contains datablock(s) I. DOI: 10.1107/S2056989016001870/wm5266Isup2.hkl

CCDC reference: 1451089

Additional supporting information:  crystallographic information; 3D view; checkCIF report

## Figures and Tables

**Table 1:** Hydrogen-bond geometry (Å, °)

*D*—H⋯*A*	*D*—H	H⋯*A*	*D*⋯*A*	*D*—H⋯*A*
O1*A*—H1*O*1⋯Cl3*B*	0.82 (1)	2.25 (2)	2.9670 (14)	146 (2)
O1*A*—H2*O*1⋯Cl1*B*	0.83 (1)	2.22 (1)	3.0227 (14)	163 (2)

**Table 2:** Experimental details

Crystal data	
Chemical formula	[CrCl(C_10_H_8_N_2_)_2_(H_2_O)][ZnCl_4_]
*M*_r_	625.00
Crystal system, space group	Monoclinic, *P*2_1_/*c*
Temperature (K)	243
*a*, *b*, *c* (Å)	9.6110 (19), 14.837 (3), 17.283 (4)
β (°)	94.93 (3)
*V* (Å^3^)	2455.4 (9)
*Z*	4
Radiation type	Synchrotron, λ = 0.600 Å
μ (mm^−1^)	1.24
Crystal size (mm)	0.15 × 0.11 × 0.09

Data collection	
Diffractometer	ADSC Q210 CCD area-detector
Absorption correction	Empirical (using intensity measurements) (*HKL-3000SM *SCALEPACK**; Otwinowski & Minor, 1997 ▸)
*T*_min_, *T*_max_	0.836, 0.897
No. of measured, independent and observed [*I* > 2σ(*I*)] reflections	13540, 7034, 6781
*R*_int_	0.012
(sin θ/λ)_max_ (Å^−1^)	0.704

Refinement	
*R*[*F* ^2^ > 2σ(*F* ^2^)], *wR*(*F* ^2^), *S*	0.027, 0.074, 1.06
No. of reflections	7034
No. of parameters	295
No. of restraints	3
H-atom treatment	H atoms treated by a mixture of independent and constrained refinement
Δρ_max_, Δρ_min_ (e Å^−3^)	0.69, −0.46

Computer programs: *PAL BL2D-SMDC* (Shin *et al.*, 2016 ▸), *HKL-3000SM* (Otwinowski & Minor, 1997 ▸), *SHELXT2014* (Sheldrick, 2015*a* ▸), *SHELXL2014* (Sheldrick, 2015*b* ▸), *DIAMOND* (Putz & Brandenburg, 2014 ▸) and *publCIF* (Westrip, 2010 ▸).

## supplementary crystallographic information

### Crystal data

**Table:**

[CrCl(C_10_H_8_N_2_)_2_(H_2_O)][ZnCl_4_]	*F*(000) = 1252
*M**_r_* = 625.00	*D*_x_ = 1.691 Mg m^−^^3^
Monoclinic, *P*2_1_/*c*	Synchrotron radiation, λ = 0.600 Å
*a* = 9.6110 (19) Å	Cell parameters from 61381 reflections
*b* = 14.837 (3) Å	θ = 0.3–33.8°
*c* = 17.283 (4) Å	µ = 1.24 mm^−^^1^
β = 94.93 (3)°	*T* = 243 K
*V* = 2455.4 (9) Å^3^	Block, orange
*Z* = 4	0.15 × 0.11 × 0.09 mm

### Data collection

**Table:**

ADSC Q210 CCD area-detector diffractometer	6781 reflections with *I* > 2σ(*I*)
Radiation source: PLSII 2D bending magnet	*R*_int_ = 0.012
ω scan	θ_max_ = 25.0°, θ_min_ = 2.3°
Absorption correction: empirical (using intensity measurements) (*HKL-3000SM *SCALEPACK**; Otwinowski & Minor, 1997)	*h* = −13→13
*T*_min_ = 0.836, *T*_max_ = 0.897	*k* = −20→20
13540 measured reflections	*l* = −24→24
7034 independent reflections	

### Refinement

**Table:**

Refinement on *F*^2^	3 restraints
Least-squares matrix: full	Hydrogen site location: mixed
*R*[*F*^2^ > 2σ(*F*^2^)] = 0.027	H atoms treated by a mixture of independent and constrained refinement
*wR*(*F*^2^) = 0.074	*w* = 1/[σ^2^(*F*_o_^2^) + (0.0324*P*)^2^ + 1.5337*P*] where *P* = (*F*_o_^2^ + 2*F*_c_^2^)/3
*S* = 1.06	(Δ/σ)_max_ = 0.002
7034 reflections	Δρ_max_ = 0.69 e Å^−^^3^
295 parameters	Δρ_min_ = −0.46 e Å^−^^3^

### Special details

**Table:**

Geometry. All e.s.d.'s (except the e.s.d. in the dihedral angle between two l.s. planes) are estimated using the full covariance matrix. The cell e.s.d.'s are taken into account individually in the estimation of e.s.d.'s in distances, angles and torsion angles; correlations between e.s.d.'s in cell parameters are only used when they are defined by crystal symmetry. An approximate (isotropic) treatment of cell e.s.d.'s is used for estimating e.s.d.'s involving l.s. planes.

### Fractional atomic coordinates and isotropic or equivalent isotropic displacement parameters (Å^2^)

**Table:**

	*x*	*y*	*z*	*U*_iso_*/*U*_eq_	
Cr1A	0.53108 (2)	0.78814 (2)	0.64562 (2)	0.01992 (5)	
Cl1A	0.43307 (5)	0.91296 (3)	0.69449 (3)	0.03711 (9)	
O1A	0.61596 (14)	0.75652 (9)	0.75105 (7)	0.0339 (2)	
H1O1	0.605 (2)	0.7882 (13)	0.7887 (9)	0.041*	
H2O1	0.673 (2)	0.7155 (11)	0.7622 (12)	0.041*	
N1A	0.69545 (12)	0.86532 (8)	0.61523 (7)	0.0249 (2)	
N2A	0.46774 (12)	0.81594 (8)	0.53187 (6)	0.0216 (2)	
N3A	0.60919 (12)	0.66584 (8)	0.61540 (7)	0.0223 (2)	
N4A	0.36310 (12)	0.70560 (8)	0.66145 (7)	0.0228 (2)	
C1A	0.80607 (17)	0.88963 (12)	0.66343 (10)	0.0346 (3)	
H1A	0.8201	0.8618	0.7123	0.042*	
C2A	0.90005 (18)	0.95447 (13)	0.64336 (11)	0.0401 (4)	
H2A	0.9773	0.9699	0.6779	0.048*	
C3A	0.87873 (18)	0.99611 (12)	0.57200 (11)	0.0384 (4)	
H3A	0.9395	1.0418	0.5581	0.046*	
C4A	0.76646 (17)	0.96983 (10)	0.52087 (10)	0.0321 (3)	
H4A	0.7518	0.9963	0.4714	0.038*	
C5A	0.67643 (14)	0.90394 (9)	0.54406 (8)	0.0230 (2)	
C6A	0.55321 (14)	0.87184 (9)	0.49560 (8)	0.0217 (2)	
C7A	0.52507 (16)	0.89530 (11)	0.41818 (8)	0.0288 (3)	
H7A	0.5848	0.9346	0.3941	0.035*	
C8A	0.40756 (18)	0.85998 (12)	0.37691 (9)	0.0344 (3)	
H8A	0.3867	0.8752	0.3244	0.041*	
C9A	0.32154 (17)	0.80242 (13)	0.41329 (9)	0.0349 (3)	
H9A	0.2419	0.7775	0.3860	0.042*	
C10A	0.35474 (15)	0.78200 (11)	0.49104 (9)	0.0288 (3)	
H10A	0.2959	0.7430	0.5160	0.035*	
C11A	0.73950 (15)	0.65088 (11)	0.59628 (9)	0.0294 (3)	
H11A	0.7995	0.7003	0.5918	0.035*	
C12A	0.78853 (17)	0.56522 (13)	0.58291 (10)	0.0375 (3)	
H12A	0.8809	0.5566	0.5704	0.045*	
C13A	0.7009 (2)	0.49304 (12)	0.58806 (12)	0.0427 (4)	
H13A	0.7320	0.4344	0.5784	0.051*	
C14A	0.56573 (19)	0.50750 (11)	0.60769 (12)	0.0384 (4)	
H14A	0.5044	0.4587	0.6117	0.046*	
C15A	0.52214 (15)	0.59467 (9)	0.62131 (8)	0.0252 (2)	
C16A	0.38330 (14)	0.61734 (10)	0.64610 (8)	0.0250 (2)	
C17A	0.27915 (18)	0.55411 (12)	0.65474 (11)	0.0363 (3)	
H17A	0.2935	0.4931	0.6429	0.044*	
C18A	0.15382 (17)	0.58197 (13)	0.68100 (11)	0.0389 (4)	
H18A	0.0825	0.5399	0.6874	0.047*	
C19A	0.13444 (16)	0.67164 (13)	0.69771 (10)	0.0363 (3)	
H19A	0.0505	0.6916	0.7161	0.044*	
C20A	0.24126 (16)	0.73197 (11)	0.68689 (9)	0.0304 (3)	
H20A	0.2280	0.7933	0.6977	0.036*	
Zn1B	0.89694 (2)	0.75938 (2)	0.89811 (2)	0.02714 (5)	
Cl1B	0.84654 (4)	0.63500 (3)	0.82027 (2)	0.03349 (8)	
Cl2B	1.02613 (4)	0.85526 (3)	0.83270 (3)	0.04086 (10)	
Cl3B	0.68071 (4)	0.82280 (2)	0.91209 (2)	0.02954 (8)	
Cl4B	1.00317 (4)	0.72294 (3)	1.01399 (2)	0.03789 (9)	

### Atomic displacement parameters (Å^2^)

**Table:**

	*U*^11^	*U*^22^	*U*^33^	*U*^12^	*U*^13^	*U*^23^
Cr1A	0.02060 (10)	0.01995 (10)	0.01930 (10)	−0.00291 (7)	0.00227 (7)	0.00242 (7)
Cl1A	0.0480 (2)	0.02427 (16)	0.0410 (2)	0.00339 (14)	0.01510 (16)	−0.00026 (14)
O1A	0.0408 (6)	0.0361 (6)	0.0234 (5)	0.0083 (5)	−0.0045 (4)	0.0013 (4)
N1A	0.0238 (5)	0.0250 (5)	0.0260 (5)	−0.0075 (4)	0.0025 (4)	−0.0005 (4)
N2A	0.0197 (5)	0.0236 (5)	0.0215 (5)	−0.0012 (4)	0.0018 (4)	0.0033 (4)
N3A	0.0216 (5)	0.0235 (5)	0.0218 (5)	−0.0011 (4)	0.0023 (4)	0.0023 (4)
N4A	0.0210 (5)	0.0249 (5)	0.0232 (5)	−0.0028 (4)	0.0047 (4)	0.0043 (4)
C1A	0.0305 (7)	0.0401 (8)	0.0326 (7)	−0.0130 (6)	−0.0008 (6)	−0.0040 (6)
C2A	0.0319 (8)	0.0431 (9)	0.0460 (9)	−0.0179 (7)	0.0067 (7)	−0.0142 (7)
C3A	0.0359 (8)	0.0313 (7)	0.0504 (9)	−0.0155 (6)	0.0184 (7)	−0.0104 (7)
C4A	0.0340 (7)	0.0257 (6)	0.0385 (8)	−0.0075 (6)	0.0146 (6)	0.0009 (6)
C5A	0.0234 (6)	0.0192 (5)	0.0274 (6)	−0.0023 (4)	0.0081 (5)	−0.0008 (5)
C6A	0.0231 (6)	0.0195 (5)	0.0232 (6)	0.0018 (4)	0.0061 (4)	0.0021 (4)
C7A	0.0322 (7)	0.0305 (7)	0.0247 (6)	0.0046 (5)	0.0082 (5)	0.0072 (5)
C8A	0.0363 (8)	0.0454 (9)	0.0216 (6)	0.0092 (7)	0.0016 (5)	0.0049 (6)
C9A	0.0285 (7)	0.0476 (9)	0.0272 (7)	0.0011 (6)	−0.0052 (5)	0.0006 (6)
C10A	0.0230 (6)	0.0349 (7)	0.0278 (7)	−0.0039 (5)	−0.0013 (5)	0.0038 (5)
C11A	0.0233 (6)	0.0355 (7)	0.0299 (7)	0.0006 (5)	0.0046 (5)	0.0021 (6)
C12A	0.0289 (7)	0.0428 (9)	0.0411 (8)	0.0104 (6)	0.0041 (6)	0.0008 (7)
C13A	0.0413 (9)	0.0308 (8)	0.0556 (11)	0.0127 (7)	0.0012 (8)	−0.0021 (7)
C14A	0.0381 (8)	0.0240 (7)	0.0532 (10)	0.0004 (6)	0.0038 (7)	0.0014 (7)
C15A	0.0257 (6)	0.0228 (6)	0.0269 (6)	−0.0019 (5)	0.0010 (5)	0.0024 (5)
C16A	0.0245 (6)	0.0240 (6)	0.0266 (6)	−0.0042 (5)	0.0020 (5)	0.0036 (5)
C17A	0.0336 (8)	0.0289 (7)	0.0467 (9)	−0.0109 (6)	0.0054 (6)	0.0025 (6)
C18A	0.0284 (7)	0.0449 (9)	0.0438 (9)	−0.0143 (7)	0.0054 (6)	0.0082 (7)
C19A	0.0242 (6)	0.0481 (9)	0.0377 (8)	−0.0042 (6)	0.0090 (6)	0.0070 (7)
C20A	0.0255 (6)	0.0336 (7)	0.0332 (7)	0.0001 (5)	0.0091 (5)	0.0031 (6)
Zn1B	0.02322 (8)	0.02820 (9)	0.02963 (9)	0.00186 (6)	0.00013 (6)	0.00117 (6)
Cl1B	0.03265 (17)	0.02719 (16)	0.04083 (19)	0.00538 (13)	0.00427 (14)	−0.00457 (14)
Cl2B	0.03470 (19)	0.0391 (2)	0.0483 (2)	−0.00872 (16)	0.00058 (16)	0.01081 (17)
Cl3B	0.02800 (16)	0.02993 (16)	0.03030 (16)	0.00691 (12)	0.00020 (12)	−0.00440 (13)
Cl4B	0.02760 (17)	0.0549 (2)	0.03070 (18)	0.00812 (16)	−0.00009 (13)	0.00489 (16)

### Geometric parameters (Å, º)

**Table:**

Cr1A—O1A	1.9876 (12)	C7A—H7A	0.9400
Cr1A—N3A	2.0485 (13)	C8A—C9A	1.377 (3)
Cr1A—N2A	2.0494 (12)	C8A—H8A	0.9400
Cr1A—N1A	2.0556 (12)	C9A—C10A	1.388 (2)
Cr1A—N4A	2.0632 (12)	C9A—H9A	0.9400
Cr1A—Cl1A	2.2732 (6)	C10A—H10A	0.9400
O1A—H1O1	0.817 (9)	C11A—C12A	1.382 (2)
O1A—H2O1	0.831 (9)	C11A—H11A	0.9400
N1A—C1A	1.3425 (19)	C12A—C13A	1.370 (3)
N1A—C5A	1.3551 (18)	C12A—H12A	0.9400
N2A—C10A	1.3413 (18)	C13A—C14A	1.387 (3)
N2A—C6A	1.3580 (16)	C13A—H13A	0.9400
N3A—C11A	1.3409 (18)	C14A—C15A	1.386 (2)
N3A—C15A	1.3564 (17)	C14A—H14A	0.9400
N4A—C20A	1.3440 (19)	C15A—C16A	1.475 (2)
N4A—C16A	1.3534 (19)	C16A—C17A	1.3893 (19)
C1A—C2A	1.384 (2)	C17A—C18A	1.386 (3)
C1A—H1A	0.9400	C17A—H17A	0.9400
C2A—C3A	1.379 (3)	C18A—C19A	1.378 (3)
C2A—H2A	0.9400	C18A—H18A	0.9400
C3A—C4A	1.390 (3)	C19A—C20A	1.387 (2)
C3A—H3A	0.9400	C19A—H19A	0.9400
C4A—C5A	1.3873 (18)	C20A—H20A	0.9400
C4A—H4A	0.9400	Zn1B—Cl4B	2.2348 (7)
C5A—C6A	1.470 (2)	Zn1B—Cl2B	2.2550 (6)
C6A—C7A	1.3865 (19)	Zn1B—Cl1B	2.3104 (6)
C7A—C8A	1.386 (2)	Zn1B—Cl3B	2.3127 (6)
			
O1A—Cr1A—N3A	83.92 (5)	C8A—C7A—C6A	118.98 (14)
O1A—Cr1A—N2A	172.59 (5)	C8A—C7A—H7A	120.5
N3A—Cr1A—N2A	91.08 (5)	C6A—C7A—H7A	120.5
O1A—Cr1A—N1A	95.85 (6)	C9A—C8A—C7A	119.61 (14)
N3A—Cr1A—N1A	96.99 (5)	C9A—C8A—H8A	120.2
N2A—Cr1A—N1A	79.29 (5)	C7A—C8A—H8A	120.2
O1A—Cr1A—N4A	90.14 (6)	C8A—C9A—C10A	118.70 (15)
N3A—Cr1A—N4A	79.41 (5)	C8A—C9A—H9A	120.6
N2A—Cr1A—N4A	94.32 (5)	C10A—C9A—H9A	120.6
N1A—Cr1A—N4A	172.67 (5)	N2A—C10A—C9A	122.41 (14)
O1A—Cr1A—Cl1A	90.13 (4)	N2A—C10A—H10A	118.8
N3A—Cr1A—Cl1A	171.51 (3)	C9A—C10A—H10A	118.8
N2A—Cr1A—Cl1A	95.39 (4)	N3A—C11A—C12A	122.18 (15)
N1A—Cr1A—Cl1A	89.61 (4)	N3A—C11A—H11A	118.9
N4A—Cr1A—Cl1A	94.61 (4)	C12A—C11A—H11A	118.9
Cr1A—O1A—H1O1	121.6 (15)	C13A—C12A—C11A	119.19 (15)
Cr1A—O1A—H2O1	126.6 (15)	C13A—C12A—H12A	120.4
H1O1—O1A—H2O1	111.3 (18)	C11A—C12A—H12A	120.4
C1A—N1A—C5A	119.17 (13)	C12A—C13A—C14A	119.26 (16)
C1A—N1A—Cr1A	125.19 (11)	C12A—C13A—H13A	120.4
C5A—N1A—Cr1A	114.89 (9)	C14A—C13A—H13A	120.4
C10A—N2A—C6A	118.75 (12)	C15A—C14A—C13A	119.28 (16)
C10A—N2A—Cr1A	126.12 (10)	C15A—C14A—H14A	120.4
C6A—N2A—Cr1A	115.10 (9)	C13A—C14A—H14A	120.4
C11A—N3A—C15A	119.01 (13)	N3A—C15A—C14A	121.08 (14)
C11A—N3A—Cr1A	125.45 (10)	N3A—C15A—C16A	115.07 (12)
C15A—N3A—Cr1A	115.36 (9)	C14A—C15A—C16A	123.80 (14)
C20A—N4A—C16A	119.31 (12)	N4A—C16A—C17A	121.03 (14)
C20A—N4A—Cr1A	125.76 (11)	N4A—C16A—C15A	115.22 (12)
C16A—N4A—Cr1A	114.91 (9)	C17A—C16A—C15A	123.74 (14)
N1A—C1A—C2A	122.03 (16)	C18A—C17A—C16A	119.22 (16)
N1A—C1A—H1A	119.0	C18A—C17A—H17A	120.4
C2A—C1A—H1A	119.0	C16A—C17A—H17A	120.4
C3A—C2A—C1A	119.05 (16)	C19A—C18A—C17A	119.58 (15)
C3A—C2A—H2A	120.5	C19A—C18A—H18A	120.2
C1A—C2A—H2A	120.5	C17A—C18A—H18A	120.2
C2A—C3A—C4A	119.36 (14)	C18A—C19A—C20A	118.69 (15)
C2A—C3A—H3A	120.3	C18A—C19A—H19A	120.7
C4A—C3A—H3A	120.3	C20A—C19A—H19A	120.7
C5A—C4A—C3A	118.90 (15)	N4A—C20A—C19A	122.16 (15)
C5A—C4A—H4A	120.6	N4A—C20A—H20A	118.9
C3A—C4A—H4A	120.6	C19A—C20A—H20A	118.9
N1A—C5A—C4A	121.43 (14)	Cl4B—Zn1B—Cl2B	111.91 (2)
N1A—C5A—C6A	114.80 (11)	Cl4B—Zn1B—Cl1B	112.67 (2)
C4A—C5A—C6A	123.76 (13)	Cl2B—Zn1B—Cl1B	107.99 (2)
N2A—C6A—C7A	121.55 (13)	Cl4B—Zn1B—Cl3B	110.50 (3)
N2A—C6A—C5A	115.13 (11)	Cl2B—Zn1B—Cl3B	109.52 (2)
C7A—C6A—C5A	123.32 (13)	Cl1B—Zn1B—Cl3B	103.92 (2)
			
C5A—N1A—C1A—C2A	1.4 (2)	C15A—N3A—C11A—C12A	−0.5 (2)
Cr1A—N1A—C1A—C2A	−168.14 (13)	Cr1A—N3A—C11A—C12A	174.38 (12)
N1A—C1A—C2A—C3A	0.7 (3)	N3A—C11A—C12A—C13A	1.1 (3)
C1A—C2A—C3A—C4A	−2.3 (3)	C11A—C12A—C13A—C14A	−0.9 (3)
C2A—C3A—C4A—C5A	1.8 (2)	C12A—C13A—C14A—C15A	0.3 (3)
C1A—N1A—C5A—C4A	−1.9 (2)	C11A—N3A—C15A—C14A	−0.2 (2)
Cr1A—N1A—C5A—C4A	168.70 (11)	Cr1A—N3A—C15A—C14A	−175.58 (13)
C1A—N1A—C5A—C6A	179.33 (13)	C11A—N3A—C15A—C16A	177.30 (12)
Cr1A—N1A—C5A—C6A	−10.10 (15)	Cr1A—N3A—C15A—C16A	1.95 (15)
C3A—C4A—C5A—N1A	0.3 (2)	C13A—C14A—C15A—N3A	0.3 (3)
C3A—C4A—C5A—C6A	178.97 (14)	C13A—C14A—C15A—C16A	−177.00 (16)
C10A—N2A—C6A—C7A	0.9 (2)	C20A—N4A—C16A—C17A	1.3 (2)
Cr1A—N2A—C6A—C7A	178.79 (10)	Cr1A—N4A—C16A—C17A	179.64 (12)
C10A—N2A—C6A—C5A	−178.43 (13)	C20A—N4A—C16A—C15A	−177.81 (13)
Cr1A—N2A—C6A—C5A	−0.56 (14)	Cr1A—N4A—C16A—C15A	0.51 (15)
N1A—C5A—C6A—N2A	7.06 (17)	N3A—C15A—C16A—N4A	−1.62 (18)
C4A—C5A—C6A—N2A	−171.70 (13)	C14A—C15A—C16A—N4A	175.83 (15)
N1A—C5A—C6A—C7A	−172.27 (13)	N3A—C15A—C16A—C17A	179.28 (14)
C4A—C5A—C6A—C7A	9.0 (2)	C14A—C15A—C16A—C17A	−3.3 (2)
N2A—C6A—C7A—C8A	−0.6 (2)	N4A—C16A—C17A—C18A	−1.3 (2)
C5A—C6A—C7A—C8A	178.66 (14)	C15A—C16A—C17A—C18A	177.74 (15)
C6A—C7A—C8A—C9A	−0.1 (2)	C16A—C17A—C18A—C19A	0.3 (3)
C7A—C8A—C9A—C10A	0.6 (3)	C17A—C18A—C19A—C20A	0.7 (3)
C6A—N2A—C10A—C9A	−0.4 (2)	C16A—N4A—C20A—C19A	−0.3 (2)
Cr1A—N2A—C10A—C9A	−178.06 (12)	Cr1A—N4A—C20A—C19A	−178.44 (12)
C8A—C9A—C10A—N2A	−0.3 (3)	C18A—C19A—C20A—N4A	−0.7 (3)

### Hydrogen-bond geometry (Å, º)

**Table:**

*D*—H···*A*	*D*—H	H···*A*	*D*···*A*	*D*—H···*A*
O1*A*—H1*O*1···Cl3*B*	0.82 (1)	2.25 (2)	2.9670 (14)	146 (2)
O1*A*—H2*O*1···Cl1*B*	0.83 (1)	2.22 (1)	3.0227 (14)	163 (2)
